# One-stage posterior debridement and single-segment interbody fusion for treating mono-segmental lumbar and lumbosacral spinal tuberculosis in adults following minimum 5-year follow-up

**DOI:** 10.1186/s13018-020-02005-w

**Published:** 2020-10-14

**Authors:** Zhenchao Xu, Xiyang Wang, Zheng Liu

**Affiliations:** 1 Department of Spine Surgery and Orthopaedics, Xiangya Hospital, Central South University, Changsha, China; 2 Hunan Engineering Laboratory of Advanced Artificial Osteo-Materials, Central South University, Changsha, China

**Keywords:** Posterior debridement, Mono-segmental lumbar spinal tuberculosis, Mono-segmental lumbosacral spinal tuberculosis, Single-segment fixation

## Abstract

**Background:**

To evaluate the mid-long-term outcomes of surgical management of mono-segmental lumbar and lumbosacral spinal tuberculosis (TB) in adults by one-stage posterior debridement, single-segment fixation, and titanium mesh cage interbody fusion.

**Methods:**

A total of 62 patients with mono-segmental lumbar or lumbosacral spinal tuberculosis were enrolled. One-stage posterior debridement, single-segment fixation, and titanium mesh cage interbody fusion was performed. Clinical and radiographic outcomes were compared and analyzed.

**Results:**

All patients were followed-up for an average of 75.0 ± 11.5 months and completely cured at the final follow-up. C-reactive protein (CRP) and erythrocyte sedimentation rate (ESR) returned to normal within three months postoperatively. Postoperative Japanese Orthopedic Association (JOA) score, visual analog scale (VAS) and Oswestry Disability index (ODI) were significantly improved compared with preoperative values. Bony fusion occurred after an average of 9.8 ± 2.6 months. The lordosis angle and lumbosacral angle were increased from preoperative 20.4 ± 2.9° and 14.7 ± 3.4° to postoperative 32.8 ± 3.6° and 22.4 ± 5.5°, with angle loss of 1.0 ± 0.7° and 0.8 ± 0.7° at the final follow-up, respectively. No significant differences between preoperative and postoperative adjacent segment disc height (DH) were found.

**Conclusions:**

One-stage posterior debridement, single-segment fixation, and titanium mesh cage interbody fusion represent effective and feasible treatment option for mono-segmental lumbar and lumbosacral spinal tuberculosis in adults. This approach may preserve lumbar normal motor units and decrease adjacent segment degeneration (ASD) with the advantages of minimal invasiveness and rapid postoperative rehabilitation.

## Background

According to the global TB report in 2019 by World Health Organization, TB is the second most common fatal infectious diseases. There are more than 10 million new TB cases worldwide in 2019, causing approximately 1.2 million deaths [1]. As the most common extrapulmonary TB, spinal TB accounts for approximately 50% cases of skeletal TB [2]. With the increasing rate of HIV infection and TB drug-resistant strains, the prevalence of spinal TB has recently continued to rise. The lumbar and lumbosacral spine are sites most commonly affected by spinal TB. This severe disease is accompanied by a high refractory, disability, and recurrence rate, which seriously influences the quality of patients’ life [3, 4].

Effective anti-TB drugs are still mainstay therapy for most patients with lumbar and lumbosacral spinal TB [5]. However, appropriate surgical treatment is the key to improve the cure rate. The aim of surgery is to remove lesions, improve neurological function and reconstruct spinal stability [6]. With the introduction of the spinal pedicle screw system, one-stage posterior approach has been increasingly adopted to treat lumbar and lumbosacral spinal TB by surgeons [7–9]. The rigid internal fixation system is able to prevent kyphosis progression and severe back pain caused by spinal instability. For the treatment of mono-segmental lumbar and lumbosacral spinal TB in adults, fixation including one or more normal motor units can be performed via posterior decompression and instrumentation. Although this procedure provides firm temporary stability, it also limits the activity of normal spinal motor unit and accelerates the degeneration of adjacent vertebral body [10–12]. Therefore, selection of appropriate fixed segment will determine the mid-long-term effect. In this study, we evaluated the effect of surgical treatment of mono-segmental lumbar and lumbosacral spinal TB in adults with one-stage posterior debridement, single-segment fixation, and titanium mesh cage interbody fusion after at least 5 years of follow-up.

## Methods

### Basic information

This study enrolled 62 patients with mono-segmental lumbar and lumbosacral spinal TB, who were treated by one-stage posterior debridement, single-segment fixation, and titanium mesh cage interbody fusion between January 2010 and December 2014. The inclusion criteria were as follows: (1) the lesion mainly involved a functional unit of the lumbar and lumbosacral spine (L2-S1); (2) vertebral body damage was less than 1/2 of the vertebral height so that the pedicle screw can be implanted; and (3) the paravertebral abscesses were limited to the diseased vertebrae. The exclusion criteria were as follows: (1) severe kyphosis caused by vertebral bone destruction; (2) severe osteoporosis in senile patients; (3) bone healing after tuberculous spondylitis with associated kyphosis deformity; and (4) huge paravertebral abscess or psoas abscess. The initial diagnosis of spinal TB was made based on the clinical presentations, hematologic examinations, and imaging findings, and the final diagnosis was made based on the pathological examination and tubercle bacillus culture. Patients who met the inclusion criteria were followed up for at least 5 years.

The demographic data and disease characteristics of patients are summarized in Table 1. All patients presented with constitutional symptoms such as night sweats, anorexia, weight loss, fatigue, and low back pain. Some patients also had symptoms of neurological impairment including sensory impairment and muscular weakness. All patients had elevated ESR and CRP values. Pain severity was assessed by VAS. The JOA and ODI were used to evaluate neurological function and the quality of life, respectively.

**Table 1:** Patient demographics and disease characteristics

Number of patients	Gender		Age (years)	During of symptoms (months)
	Male	Female		
62	33	29	47.5 ± 14.0	3.1 ± 1.3

All patients underwent routine imaging examinations. Plain radiograph, computed tomography (CT), and magnetic resonance imaging (MRI) were used to detect vertebral body collapse, spinal instability, bone destruction, epidural and paravertebral abscess formation, and narrowing of the intervertebral space. Lesions with segments L2/3 L3/4, L4/5, and L5/S1 occurred in 11 cases, 17 cases, 21 cases, and 13 cases, respectively. The University of California at Los Angeles (UCLA) grading scale [13] was applied to assess the ASD on radiograph.

### Preoperative procedure

All patients were treated with anti-TB drugs for 2 to 4 weeks prior to surgery, including isoniazid (300 mg/day), rifampicin (450 mg/day), and pyrazinamide (750 mg/day), and ethambutol (750 mg/day). Preoperative examinations were performed to exclude contraindications. Anemia and hypoproteinemia were corrected preoperatively, the targets for blood pressure and random blood glucose were below 140/90 mmHg and lower than 10.0 mmol/L, respectively. Surgery was performed when the ESR value returned to normal or had significantly decreased. Patients with progressive radiculopathy or cauda equine syndrome were treated with adequate anti-TB drugs. If the patients had no absolute contraindications, surgery was performed as soon as possible.

### Operation procedure

The patients were placed in a prone position under general endotracheal anesthesia. A posterior midline incision was made over the diseased vertebra, and the spinous processes, lamina, facet joints, and transverse processes were exposed. With the assistance of C-arm fluoroscopy, pedicle screws were implanted in the affected vertebrae close to the endplates in order to preserve enough debridement space. A temporary rod was stabilized on the side of less bone destruction or neurologic manifestation to avoid nerve injury during focal debridement. Then, hemi-laminectomy or laminectomy was performed on the severely damaged side of the lesion segment to expose the diseased vertebral bodies. Curettes of varying angles were used to remove the lesion focus, including sequestrum, necrotic intervertebral disc, tuberculous granuloma, and caseous necrosis. Pus and necrotic tissue were eliminated by negative pressure washing via a soft silicone tube which was placed deep into the lesion. The same procedure was performed on the other side, if necessary. The bone surfaces of the vertebral body were repaired as bone graft beds, and autogenous bone particles from healthy lamina and spinous process packed in one or two shaped titanium mesh cages were implanted into the front 2/3 of the intervertebral to reconstruct the anterior middle column. Then, the opposite rod was installed, and compression was carried out after placement of interbody titanium mesh. Streptomycin (0.1 g) and isoniazid (0.3 g) were applied locally in the focus area, and the incision was closed in layers after a drainage tube was placed. The specimens were collected for mycobacterium culture and histopathological examination.

### Postoperative procedure

Routine antibiotics were used to prevent infection. All patients received nutritional improvement and support treatment. Wound dressings were changed regularly, and the drainage tube was removed when the volume of drainage was less than 20 ml in 24 h. The patients continued with the anti-TB treatment regimen for 12 to 18 months. Routine blood test, liver function test, ESR and CRP were performed to monitor adverse reactions and to evaluate drug efficacy. After strict bed rest for 4 weeks, the patients gradually began to walk with the help of external braces for 3 months. Clinical and radiologic examinations were performed every 3 months in the first year after surgery and then every 6 months thereafter.

### Follow-up evaluation

Lumbar anterolateral radiograph and CT were performed to assess the placement of graft and internal fixation. CT was used to evaluate bone healing according to the radiologic criteria by Lee et al. [14]. The following indexes were recorded preoperatively, postoperatively, and during the follow-up: (1) ESR and CRP; (2) neurological status according to JOA; (3) ODI and VAS; (4) adjacent segment (DH); and (5) lordosis angle and lumbosacral angle.

### Statistical analysis

Statistical analyses were performed using the SPSS 20.0 software. The clinical and radiographic data between preoperative and postoperative were compared using paired *t* test. Discrepancy of the normal distribution was evaluated using the rank sum test. *P* value of less than 0.05 was considered statistically significant.

## Results

### Clinical outcomes

Tuberculous granulomas or caseous necrosis was confirmed by pathological examination of the surgical specimens. The average follow-up time was 75.0 ± 11.5 months. All patients were diagnosed with lumbar or lumbosacral spinal TB and achieved clinically cure. Preoperative CRP and ESR were 43.5 ± 14.9 mg/L and 69.1 ± 17.8 mm/h, respectively. The values returned to normal at three months postoperatively. For patients with preoperative neurological dysfunction, neurological function improved to varying degrees after surgery. The JOA improved from preoperative 18.3 ± 3.7 to 26.9 ± 2.2 at the final follow-up (*P* < 0.05). The VAS and ODI values were 6.9 ± 1.1 and 42.6 ± 6.2 preoperatively and significantly decreased to 1.0 ± 0.8 and 10.2 ± 1.6 at the last follow-up, respectively, (*P* < 0.05) (Table 2).

**Table 2:** Clinical outcomes of patients

CRP (mg/L)			ESR (mm/h)			JOA		VAS		ODI		Follow-up (months)
Pre	TMP	FFU	Pre	TMP	FFU	Pre	FFU	Pre	FFU	Pre	FFU	
43.5 ± 14.9	4.4 ± 1.2^*^	2.0 ± 0.6^*^	69.1 ± 17.8	12.0 ± 3.1^*^	4.5 ± 1.7^*^	18.3 ± 3.7	26.9 ± 2.2^*^	6.9 ± 1.1	1.0 ± 0.8^*^	42.6 ± 6.2	10.2 ± 1.6^*^	75.0 ± 11.5

*Pre* preoperative, *TMP* three months postoperative, *FFU* final follow-up

*Analyzed by paired *t* test, compared with preoperatively, *P* < 0.05

### Radiographic outcomes

**Fig. 1:**
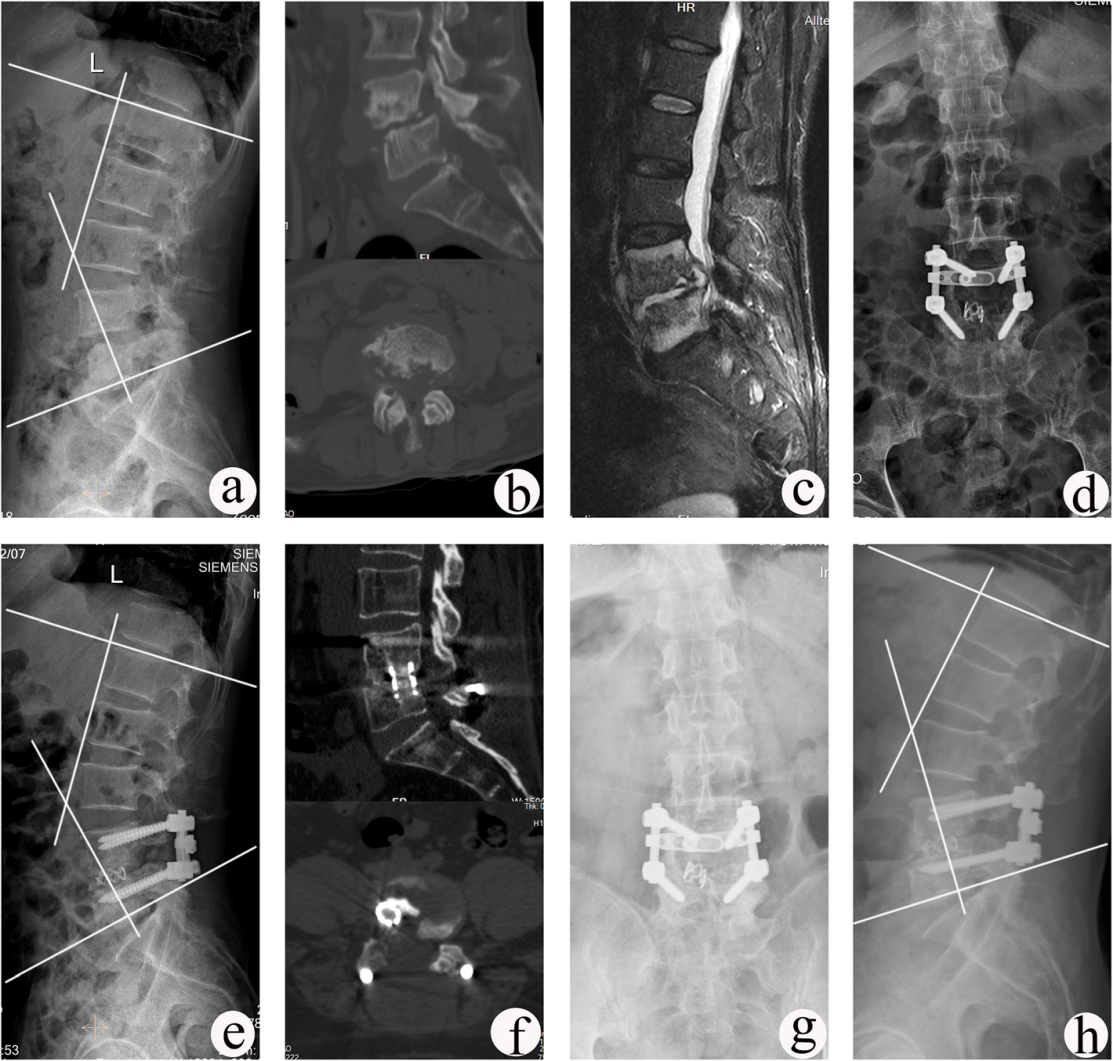
A 61-year-old male with L4–L5 TB underwent one-stage posterior debridement, titanium mesh cage bone grafting and single-segment fixation. **a**–**c** Preoperative images showing that the lesion was located at L4–L5 with a lordosis angle of 26°. **d**, **e** Postoperative X-ray demonstrating correction of the deformity (lordosis angle was 40°). **f** CT images showing satisfactory bone fusion at 9 months. **g**, **h** X-ray images displaying good internal fixation position and solid bone fusion, with no loss of lordosis angle and no change in adjacent segment DH throughout 66 months of follow-up

**Fig. 2:**
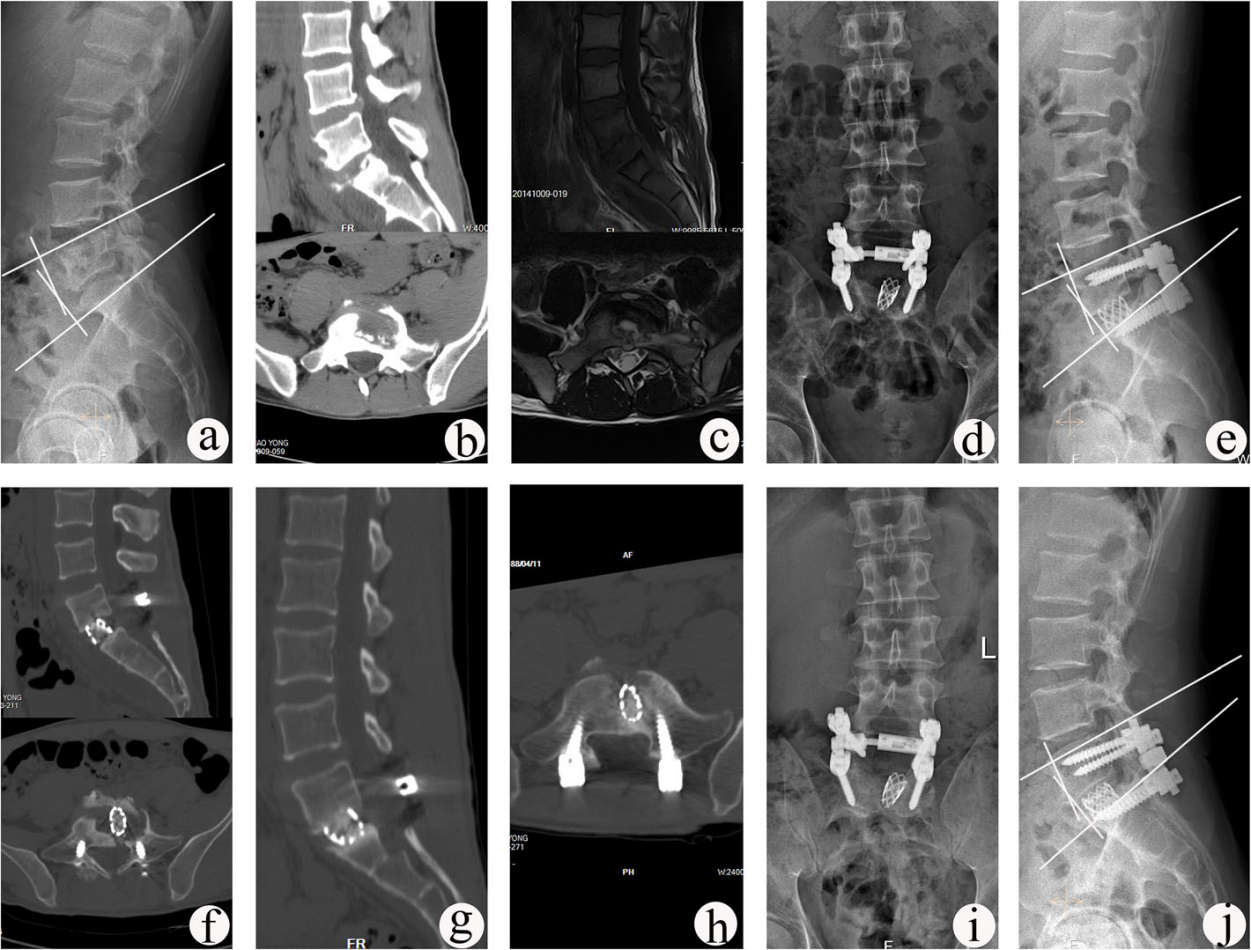
A 26-year-old male with L5–S1 TB underwent one-stage posterior debridement, titanium mesh cage bone grafting and single-segment fixation. **a**–**c** Preoperative images show lesions with lumbosacral angle of 12° and upper DH of 10.7 mm. **d**–**f** Postoperative X-ray demonstrating correction of the deformity (lumbosacral angle was 18°) and CT findings showing that the titanium mesh cage with autogenous bone particles was implanted into vertebral body. **g**, **h** CT images showing satisfactory bone fusion at 12 months after surgery. **i**, **j** X-ray displaying good internal fixation position and solid bone fusion, with the lumbosacral angle of 17° and the upper DH of 10.5 mm at the follow-up period of 81 months

The lordosis angle and lumbosacral angle increased from preoperative 20.4 ± 2.9° and 14.7 ± 3.4° to 32.8 ± 3.6° and 22.4 ± 5.5° immediately postoperative, resulting in angle loss of 1.0 ± 0.7° and 0.8 ± 0.7°at the final follow-up, respectively. The lordosis angle or lumbosacral angle was significantly improved immediately postoperative and at the last visit, compared with preoperatively measurements (*P* < 0.05). The mean fusion time was 9.8 ± 2.6 months (Table 3). There was no significant difference between preoperative and postoperative adjacent segment DH (Table 4). According to the UCLA grading scale, 52 patients had grade I ASD and 10 patients had grade II ASD preoperatively. At the final follow-up, 8 patients progressed from grade I ASD to grade II ASD, and 2 patients from grade II ASD to grade III ASD. The average rates of titanium mesh cage subsidence measured at the site of bone graft fusion were 0.8 ± 0.2 mm, 0.9 ± 0.1 mm, 1.2 ± 0.2 mm, and 1.1 ± 0.2 mm in patients with segments L2/3 L3/4, L4/5, and L5/S1, respectively. All patients achieved satisfactory bone graft fusion. No non-union, pseudoarthrosis, loosening or fracture of instruments occurred at the last visit (Figs. 1 and 2).

**Table 3:** Radiographic outcomes of patients

lordosis angle (°)					lumbosacral angle (°)					Fusion time (months)
Pre	Post	FFU	Angle lost	Correction rate (%)	Pre	Post	FFU	Angle lost	Correction rate (%)	
20.4 ± 2.9	32.8 ± 3.6^*^	31.8 ± 3.5^*^	1.0 ± 0.7	37.8 ± 4.5	14.7 ± 3.4	22.4 ± 5.5^*^	21.5 ± 5.0^*^	0.8 ± 0.7	34.1 ± 6.1	9.8 ± 2.6

Lordosis angle: 49 cases with a local lumbar lordosis angle in lesions

Lumbosacral angle: 13 cases with a local lumbosacral lordosis angle in lesions

*Pre* preoperative, *Post* postoperative immediately, *FFU* final follow-up

*Analyzed by paired *t* test, compared with preoperatively, *P* < 0.05

**Table 4:** The change of adjacent segment DH

	L2/3		L3/4		L4/5		L5/S1	
	Pre	FFU	Pre	FFU	Pre	FFU	Pre	FFU
Upper DH (mm)	8.8 ± 0.4	8.6 ± 0.3	8.6 ± 0.4	8.4 ± 0.3	10.3 ± 0.6	10.0 ± 0.5	11.1 ± 0.5	10.8 ± 0.4
Lower DH (mm)	10.1 ± 0.4	9.8 ± 0.4	10.7 ± 0.6	10.6 ± 0.6	9.7 ± 0.6	9.5 ± 0.5	/	/

*Pre* preoperative, *FFU* final follow-up

### Complications

Superficial wound infection occurred in nine patients including 2 patients with *Staphylococcus aureus*, 1 patient with *E. coli*, and the 6 patients with negative bacterial culture results. The infection was cured by antibiotics. Four patients experienced hypostatic pneumonia and were treated with sensitive antibiotics according to the results of sputum culture. Local abscess recurrence due to irregular administration of anti-TB drugs occurred in one patient, who was treated by catheter drainage through minimally invasive incision and regular chemotherapy. No operative mortality or permanent nerve injury occurred.

## Discussion

The local anatomical structures of the lumbar region are complex and adjacent to many vital organs. Lumbar stability is maintained by the combined effects of the vertebrae, intervertebral discs, muscle groups, and ligaments in the face of strong pressure and shear stress. Among all spinal regions, lumbar and lumbosacral segments subject to the largest load and exhibit the greatest mobility. Lumbar and lumbosacral spinal TB has the characteristics of insidious onset and atypical symptoms at early stages. Patients often present with low back pain and are easily misdiagnosed with degenerative spinal diseases such as lumbar disc herniation, lumbar spinal stenosis and osteoporosis [15]. With progression of the disease, sequestrum and abscesses can lead to vertebral collapse, spinal instability, kyphosis deformity, and changes in normal physiological curvature and load biomechanics, or invade the spinal canal to compress the nerves, resulting in nerve damage or even paralysis [16].

There are many surgical approaches for treating lumbar and lumbosacral spinal TB [17–20]. The posterior approach is associated with some advantages such as lesion removal, and simultaneous intervertebral bone graft and fixation through one incision without position changing. Additionally, pedicle screws can effectively correct the kyphosis via the posterior approach, thus restoring the normal physiological lordosis of the lumbar segment. Furthermore, the posterior approach is less invasive and avoids possible damage to the large vessels, nerves, or vital anatomical structures. Therefore, many surgeons recommend posterior-only approach for lumbar and lumbosacral spinal TB, which achieves good curative effects [7–9, 20]. The rationale behind the posterior approach is removal of the sequestrum that prevents anti-TB drugs from entering the lesions. The small amount of residual lesions and abscesses can be absorbed following postoperative standardized anti-TB chemotherapy.

Previous studies have shown that the range of fixation for mono-segmental lumbar and lumbosacral spinal TB in adults involves one to multiple normal motion units above and below the lesion via posterior debridement, decompression, and instrumentation. Although multi-segment fixation provides strong temporary stability, it sacrifices the motion of the fixed segments and accelerates degeneration of adjacent segments [21, 22]. Since the lumbar region requires greater mobility than the rest of vertebral column, adopting a surgical approach with minimally invasion and less damage to vertebral function is the major goal for treating mono-segmental lumbar and lumbosacral spinal TB. Previous studies have reported that the spinal motor unit remains essentially unchanged following single-segment fixation with pedicle screws in the treatment of spinal fractures. Spinal stability in patients with fractures of spine can be achieved by mono-segment fixation [23]. For the treatment of lumbar and lumbosacral spinal TB, single-segment fixation with pedicle screw is feasible because of the reactive new bone formation in vertebrae affected by TB. The involved vertebrae often presents with sclerotic bones, resulting in higher bone mineral density than normal vertebral body. This pathological process results in stronger holding forces for pedicle screws in the involved vertebrae, compared with fracture [24]. Liu et al. [25] compared the results of mono-segment fixation versus short-segment fixation for the treatment of single-segment lumbar spinal TB and found that mono-segment fixation was more suitable since the normal motion was preserved. Xu et al. [26] reported that single-segment fixation and bone fusion was effective in maintaining the stability of the spine and retaining normal motion units. In the present study, the lordosis and lumbosacral angle increased from preoperative 20.4 ± 2.9° and 14.7 ± 3.4° to postoperative 32.8 ± 3.6° and 22.4 ± 5.5. Our findings suggest that deformity was effectively corrected. The characteristic of lumbar and lumbosacral lordosis and longitudinal arrangement of facet joints, which buffer the effect of kyphosis caused by bone destruction. Therefore, mono-segment fixation is sufficient for relatively small orthopedics. In addition, there was no significant difference between the preoperative and final adjacent segment DH, and the ASD rate was lower in this study (16.1%) than the incidence of ASD (range 21.3 to 31.9%) after lumbar fusion reported by the recent meta-analysis [27]. These results suggest that mono-segment fixation can maintain the normal motion units and to some extent, retard the degeneration of adjacent segment.

Intervertebral bone grafting is critical for maintaining long-term stability of the spine after surgery according to the three columns theory of Denis [28]. Adopting posterior single-segment fixation limited exposure space, which is not suitable for large area bone grafting. One or two shaped titanium mesh cages filled with autogenous bone particles autogenous bone particles from healthy lamina and spinous process were implanted to reconstruct the anterior and middle column. This intervertebral bone grafting method by implanting ideal titanium cage increases the contact area between the bone particles and the bone grafting surface, thus facilitating the penetration of various cytokines to promote bone metabolism and accelerate the osteogenesis. Furthermore, titanium mesh cage has the characteristics of high strength, strong support, and great friction on the contact surface, which can withstand compressive force well to prevent the bone from fracture and displacement. Furthermore, according to the specific shape of the bone defect between vertebrae, the titanium mesh can be trimmed and shaped to match the shape of the bone defect. Thus, more healthy bone can be retained, thus avoiding the decrease in spinal stability due to larger bone defects after the removal of the lesions [29]. Biomechanical studies have indicated that TB bacilli adhere weakly to the titanium materials and do not affect the bactericidal effect of anti-TB drugs [30]. In this study, we found that bone graft fusion was achieved in all patients. No deep infection, internal fixation loosening, fracture, and pseudoarticular formation occurred.

The advantages of one-stage posterior debridement, titanium mesh cage bone grafting, and single-segment fixation in the treatment of mono-segmental lumbar and lumbosacral spinal TB include as follows: (1) Removal of lesions and internal intervertebral bone fusion and instruments are achieved simultaneously through one incision, thus reducing surgical trauma and the risk of damage to vital organs; (2) Single-segment fixation preserves the normal motion of the lumbar regions, maintains spinal stability and slows down the degeneration of adjacent segments; (3) pedicle screws provides three-column fixation, and effectively restores the normal physiological curvature and height of the spine. The internal fixation is placed outside the focus to avoid infection; and (4) titanium mesh cage bone graft increases the contact area and the implant is not easy to remove, which is beneficial to bone cell metabolism and improves the bone graft fusion rate.

Because there are the individual differences in lumbar and lumbosacral spinal TB, this surgical approach also has some limitations: (1) when the focus is concentrated in the anterior column or accompanied with huge abscess or flowing abscess, the anterior approach should be selected; (2) if multiple vertebral bodies are severely damaged, a combined anterior and posterior approach is required; (3) if the patients with severe osteoporosis or kyphosis, a long posterior segment fixation is needed to maintain its stability or correct the deformity. In addition, this study is a single-center study and the sample size is relatively small. In the future, prospective studies with larger samples are required to confirm the findings of this study.

## Conclusions

One-stage posterior debridement, single-segment fixation, and titanium mesh cage interbody fusion represent effective and feasible treatment option for mono-segmental lumbar and lumbosacral spinal tuberculosis in adults. This approach may preserve lumbar normal motor units and decrease ASD with the advantages of minimal invasiveness and rapid postoperative rehabilitation.

## Data Availability

The datasets and materials generated or analyzed during the current study are available from the corresponding author on reasonable request.

## Abbreviations

ASD: Adjacent segment degeneration
CRP: C-reactive protein
CT: Computed tomography
DH: Disc height
ESR: Erythrocyte sedimentation rate
JOA: Japanese Orthopaedic Association
MRI: Magnetic resonance imaging
ODI: Oswestry Disability index
TB: Tuberculosis
UCLA: University of California at Los Angeles
VAS: Visual analog scale
